# Clinical significance of the nuclear receptor co-regulator DC-SCRIPT in breast cancer: an independent retrospective validation study

**DOI:** 10.1186/bcr2786

**Published:** 2010-12-01

**Authors:** Anieta M Sieuwerts, Marleen Ansems, Maxime P Look, Paul N Span, Vanja de Weerd, Anne van Galen, John A Foekens, Gosse J Adema, John WM Martens

**Affiliations:** 1 Department of Medical Oncology, Josephine Nefkens Institute and Cancer Genomics Centre, Erasmus Medical Center, Dr. Molewaterplein 50, Rotterdam, 3015 GE, The Netherlands; 2 Department of Tumor Immunology, Nijmegen Centre for Molecular Life Sciences, Radboud University Nijmegen Medical Centre, Geert Grooteplein 28, Nijmegen, 6525 GA, The Netherlands; 3 Department of Radiation Oncology and Department of Laboratory Medicine, Radboud University Nijmegen Medical Centre, Geert Grooteplein 32, Nijmegen, 6525 GA, The Netherlands

## Abstract

**Introduction:**

In this study we aimed to validate the prognostic value of *DC-SCRIPT *mRNA expression in a large independent breast cancer cohort. In addition, since DC-SCRIPT is a transcriptional co-regulator of nuclear receptors, we explored its prognostic value in relation to estrogen-receptor-α (*ESR1*) and -β (*ESR2*) and evaluated its predictive value for response to tamoxifen treatment.

**Methods:**

*DC-SCRIPT *mRNA levels were measured by real-time PCR in 1,505 primary invasive breast cancers and associated with outcome (disease-free survival (DFS), metastasis-free survival (MFS) and overall survival (OS)) using univariate and multivariable Cox regression analysis. Logistic and Cox regressions were used to associate *DC-SCRIPT *levels with clinical benefit and progression-free survival (PFS) for 296 patients treated with first-line systemic tamoxifen for advanced disease.

**Results:**

In univariate and multivariable analysis higher *DC-SCRIPT *levels were associated with a favorable outcome for both the entire cohort and patients with lymph node-negative (LNN) disease that did not receive adjuvant therapy (DFS, MFS and OS; all, *P *< 0.001). This association was most pronounced in small (pT1) tumors, in *ESR1*-positive tumors and in tumors with low *ESR2 *expression. For first-line endocrine therapy for advanced disease no predictive association was seen with clinical benefit or PFS.

**Conclusions:**

This study provides a higher level of evidence that *DC-SCRIPT *is indeed an independent, pure prognostic, factor for primary breast cancer and shows that *DC-SCRIPT *mRNA expression is most informative for either *ESR1*-positive and/or *ESR2*-low pT1 tumors.

## Introduction

Estrogens influence the aggressiveness of breast cancer through their cognate nuclear receptors. In particular, the estrogen receptor-alpha (ERα) (*ESR1*) - present in tumor cells of about 70% to 75% of all breast tumors - is considered crucial because of its proliferation-inducing actions and for that reason is an important target for therapy. Next to *ESR1*, a second ER exists, ERβ (*ESR2*). *ESR2 *counteracts the activity of *ESR1 *in many systems [1,2] and is also expressed in the majority of breast cancers. Apart from breast epithelial tumor cells, *ESR2 *is also expressed in adjacent infiltrating lymphocytes, fibroblasts, and endothelial cells, all of which are known to influence tumor growth [3]. However, its precise role in breast cancer progression is less well defined.

DC-SCRIPT (zinc finger protein 366 [*ZNF366*]) is a recently identified nuclear receptor co-regulator first identified in immune cells [4-6]. Nuclear receptor co-regulators are proteins that can activate or repress the transcriptional activity of nuclear receptors. DC-SCRIPT is in this respect a unique co-regulator as we have shown that it enhances the activities of the nuclear retinoic acid receptor (RAR) and peroxisome proliferator-activated receptor (PPAR) heterodimers, RARα/RXRα and PPARγ/RXRα, but represses the activities of *ESR1 *and progesterone receptor (*PGR*) [7]. We also showed that DC-SCRIPT was an independent prognostic factor, particularly for hormone receptor-positive breast cancer. This led us to postulate that the anti-proliferative effect of DC-SCRIPT in breast cancer cells could be mediated by simultaneous modulation of the activity of multiple nuclear receptors.

To provide a higher level of evidence for *DC-SCRIPT *mRNA expression as a prognostic marker, we now report on *DC-SCRIPT *expression and its significance in a retrospective validation study of 1,505 breast cancer patients with known *ESR1*, *ESR2*, and *PGR *expression levels. The primary objective of this study was to confirm the relationship between *DC-SCRIPT *mRNA levels measured in primary breast cancers and tumor aggressiveness in a much larger, independent, breast cancer cohort. The main clinical endpoints for assessing the prognostic value of *DC-SCRIPT *expression were disease-free survival (DFS), metastasis-free survival (MFS), and overall survival (OS) in lymph node-negative (LNN) patients who had not received adjuvant systemic therapy; this approach allowed us to determine tumor aggressiveness during the natural course of the disease. As DC-SCRIPT modulates ER activity, we also analyzed the prognostic value of *DC-SCRIPT *separately in tumors stratified by *ESR1 *and *ESR2 *expression. Since several co-regulators of nuclear receptors also modulate response to therapy [8,9], we also assessed, as a secondary aim of this study, the predictive value of *DC-SCRIPT *by using clinical benefit and progression-free survival (PFS) after first-line tamoxifen for advanced disease as the main endpoints.

## Materials and methods

### Patients

The protocol to study biological markers associated with disease outcome was approved by the medical ethics committee of the Erasmus Medical Center (Rotterdam, The Netherlands) (MEC 02.953). This retrospective study used 1,505 M0 (no metastasis) and 32 M1 (with metastasis) blind-coded freshly frozen primary tumor tissues of female patients with primary operable breast cancer from 1978 through 2000. The study was performed in accordance with the Code of Conduct of the Federation of Medical Scientific Societies in The Netherlands [10], and consent was not required. Wherever possible, the study has been reported in accordance with the Reporting Recommendations for Tumor Marker Prognostic Studies guidelines [11]. The primary breast tumors were from patients with detailed clinical follow-up as previously described [12-14]. ER protein status was determined by routine ligand-binding assays or enzyme immunoassays [15], and *ESR1*, *ESR2*, and *PGR *mRNA status was determined by real-time reverse transcriptase-polymerase chain reaction (RT-PCR) [14,16,17]. Follow-up, tumor staging, and response to therapy were defined by standard International Union Against Cancer (Geneva, Switzerland) classification criteria [18] and applied previously by Foekens and colleagues [19]. All 1,537 patients underwent breast-conserving lumpectomy (44%) or modified mastectomy (56%). Of the 1,505 patients included for the evaluation of tumor aggressiveness, 462 lymph node-positive patients (31%) were treated with adjuvant systemic therapy, 207 patients received hormonal therapy, 233 chemotherapy, and 22 combination therapy. Disease recurrence occurred in 836 patients, and 703 developed a distant metastasis. The median follow-up time of patients alive was 90 months (range of 4 to 260 months).

Eight hundred thirty-seven patients had no involved nodes and did not receive systemic adjuvant therapy. Of these 837 LNN patients, 383 had a disease relapse, 300 developed a distant metastasis, and 273 died during follow-up.

Of the 703 patients who developed a distant metastasis, 296 ER-positive patients, including the 32 M1 patients, received hormonal therapy as first-line therapy for advanced disease. Clinical benefit of first-line tamoxifen treatment was observed in 185 patients. Median follow-up time for treatment of advanced disease was 38 (4 to 120) months. Two hundred nineteen patients had died at the end of the follow-up. None of these patients had received prior adjuvant hormonal therapy, whereas 19% received prior adjuvant chemotherapy. A more detailed description of the patients and their therapy is given in the Supplementary materials and methods (Additional file 1). Patient and tumor characteristics combined with *DC-SCRIPT *mRNA expression and clinical outcome are listed in Table 1.

**Table 1:** Associations of *DC-SCRIPT *with clinicopathological and biological factors

Characteristic	Number of patients	Percentage^a^	*DC-SCRIPT*^b ^(reference-normalized), × 10^2^	
All patients	1,505	100%	0.69	0.73
Age, years				
≤ 40	192	13%	0.69	0.72
41-55	561	37%	0.70	0.74
56-70	498	33%	0.70	0.77
>70	254	17%	0.64	0.64
			*P *= 0.15^c^	
Menopausal status				
Premenopausal	637	42%	0.72	0.74
Postmenopausal	868	58%	0.66	0.70
			*P *= 0.06^d^	
Grade				
Poor	818	54%	0.64	0.74
Unknown	452	30%	0.71	0.68
Moderate and good	235	16%	0.80	0.70
			*P *= 0.001^e^	
Tumor size				
pT1, ≤ 2 cm	517	34%	0.81	0.84
>2 cm	988	66%	0.63	0.64
			*P *< 0.001^d^	
Lymph nodes involved				
No	837	56%	0.69	0.73
Yes	668	44%	0.68	0.75
			*P *= 0.64^d^	
*ESR1 *mRNA status^f^				
Positive, ≥0.2	1,176	78%	0.71	0.73
Negative, < 0.2	329	22%	0.61	0.66
			*P *= 0.004^c^	
*PGR *mRNA status^f^				
Positive, ≥0.1	949	63%	0.72	0.74
Negative, < 0.1	556	37%	0.61	0.66
			*P *< 0.001^c^	
*ESR2 *mRNA status^f^				
Dichotomized high, ≥0.005	741	49%	0.89	0.95
Dichotomized low, < 0.005	742	49%	0.54	0.49
			*P *< 0.001^c^	
Invasive tumor cell content^g^				
≥70%	719	48%	0.57	0.51
< 70%	786	52%	0.85	0.91
			*P *< 0.001^d^	
Histological type				
DCIS + IDC	194	13%	0.82	0.89
ILC	135	9%	0.81	0.94
IDC	810	54%	0.66	0.69
Mucinous	40	3%	0.56	0.65
Medullary	31	2%	0.69	1.18
			*P *= 0.012^e^	
Intrinsic breast cancer subtype^h^	308			
Normal-like	22	7%	1.43	1.19
ERBB2+	63	20%	0.75	0.68
Luminal A	76	25%	0.78	0.89
Luminal B	65	21%	0.56	0.36
Basal	82	27%	0.48	0.48
			*P *< 0.001^e^	

^a^Owing to missing cases, numbers do not always add up to 100%. ^b^Median level and p50 inter-quartile after normalization on the reference gene set. ^c^*P *for Spearman rank correlation test. ^d^*P *for Mann-Whitney *U *test. ^e^*P *for Kruskal-Wallis test, including a Wilcoxon-type test for trend when appropriate. ^f^With quantitative polymerase chain reaction cut point for positive versus negative *ESR1 *and *PGR*, 0.2 and 0.1, respectively, and for *ESR2 *at the median level of 0.005 (mRNA levels relative to reference gene set). ^g^Dichotomized at the median level of 70% invasive tumor cells. ^h^Intrinsic breast cancer subtypes assigned from Affymetrix microarray by hierarchical clustering of 308 lymph-node negative disease patients who did not receive systemic adjuvant treatment. DCIS, ductal carcinoma *in situ*; *DC-SCRIPT*, dendritic cell-specific transcript gene; ERBB2+, HER2neu-positive; *ESR*, estrogen receptor gene; IDC, infiltrating ductal carcinoma; ILC, infiltrating lobular carcinoma; *PGR*, progesterone receptor gene; pT1, small tumor without lymphatic/vascular invasion.

### RNA isolation and quantitative RT-PCR

Tissue processing, RNA isolation, cDNA synthesis, and quantitative RT-PCR were performed as previously described [16]. Real-time quantitative PCRs were performed in a 25-μL reaction volume in an M×3000P™ Real-Time PCR System (Agilent, Amsterdam, The Netherlands). In addition to an SYBR-based assay to detect a 129-base pair (bp) DC-SCRIPT transcript covering exon 4 to 5 (forward primer: 5'-AAAGTCAAGCATGGAGTCATG-3'; reverse primer: 5'-GCTTCTGAGAGAGGTCAAAG-3'), a commercially available Taqman Gene Expression Assay from Applied Biosystems (Nieuwerkerk aan den IJssel, The Netherlands) covering exon 3 to 4 and generating a 62-bp product was used (Hs00403536_m1, RefSeq NM_152625.1). *DC-SCRIPT *levels were readily detected with both assays, and data generated with these assays correlated significantly (Spearman's rho = 0.87; *P *< 0.0001). We therefore performed our analyses on the real-time RT-PCR data generated with the Taqman assay, which is generally considered to be more specific. Intron-spanning primer sequences for the three reference genes - that is, hydroxymethylbilane synthase (*HMBS*), hypoxanthine-guanine phospho-ribosyltransferase (*HPRT1*), and β-2-microglobulin (*B2M*) - and for *ESR1*, *ESR2*, *PGR*, and real-time PCR conditions for these SYBR-based assays were as described previously [16,17]. Forty rounds of amplification were performed, and fluorescent signals of the Taqman probe or SYBR green signal were used to generate cycle threshold (Ct) values from which mRNA expression levels were calculated. Ct values of *HPRT1 *and *B2M *were adjusted to the higher *HMBS *Ct values. Next, the expression levels of *DC-SCRIPT *were normalized against the average expression levels of the three reference genes as follows: mRNA target = 2^(mean Ct reference genes - mean Ct target) ^[16].

### Tissue processing

Primary tumor tissue was processed as described previously [16]. To assess the amount of invasive tumor cell nuclei relative to the amount of surrounding stromal cells, 5-μm sections were cut for hematoxylin-and-eosin staining before, during, and after the sections were cut for RNA isolation. Only specimens with at least 30% invasive tumor cell nuclei were included in this study.

### Data analysis and statistics

The relationship between *DC-SCRIPT *and patient and tumor characteristics was investigated with the use of non-parametric methods (Spearman rank correlations for continuous variables and Wilcoxon rank-sum for dichotomized or Kruskal-Wallis test for ordered variables). To reduce skewness, *DC-SCRIPT *levels were transformed with the Box-Cox transformation. *DC-SCRIPT *levels were dichotomized with the previously identified 66.7% high versus 33.3% low cutoff for *DC-SCRIPT *[7]. To test for an association with tumor aggressiveness and the time to progression during first-line therapy, Cox regression analysis was applied on the Box-Cox-transformed and dichotomized *DC-SCRIPT *mRNA levels. The hazard ratio (HR) and its 95% confidence interval were computed to correlate the expression levels with DFS, MFS, OS, and PFS, respectively. In multivariable analysis, Cox proportional hazards models for DFS, MFS, OS, and PFS were applied to test *DC-SCRIPT *levels added to models with traditional factors. The proportional hazards assumptions were checked with Schoenfeld residuals. The analyses were stratified if necessary. The models for DFS, MFS, and OS for LNN patients who had not received adjuvant systemic therapy included age, menopausal status, tumor size, grade, and *ESR1 *and *PGR *mRNA levels. Survival curves were generated with the method of Kaplan and Meier. The log-rank test was used to test for differences between survival curves. Logistic regression was used for the association of *DC-SCRIPT *with clinical benefit. Computations were performed with the STATA statistical package, release 11.0 (STATA Corp., College Station, TX, USA) and SPSS 15.0 (SPSS Inc., Chicago, IL, USA). All *P *values are two-sided, and a *P *value of less than 0.05 was considered statistically significant.

## Results

### Associations of *DC-SCRIPT *with clinicopathological factors and histological and intrinsic breast cancer subtypes

In analogy with our previous study, *DC-SCRIPT *mRNA expression was readily detected by quantitative RT-PCR in five normal breast tissues taken adjacent from tumor tissue and five prophylactic breast tissues (median [interquartile]: 0.063 [0.015] and 0.054 [0.035], respectively), whereas median levels were over 8-fold lower (*P *< 0.05) in 1,505 invasive breast tumors (0.0069 [0.0074]). Table 1 shows the median expression levels and interquartile ranges of *DC-SCRIPT *transcripts and relation with patient and tumor characteristics for these 1,505 patients who were evaluable for prognosis. *DC-SCRIPT *levels were positively associated with tumor grade and *ESR1*, *PGR*, and *ESR2 *steroid hormone receptor expression level and negatively associated with invasive epithelial tumor cell content and tumor size. In addition, *ESR2 *was more highly expressed in tumors with a higher percentage of stromal cells (786 tumors with 30% to 70% invasive epithelial cells), and *ESR1 *was more highly expressed in tumors with a high percentage of invasive epithelial cells (719 tumors with at least 70% invasive epithelial cells) (*P *< 0.001) (data not shown). High levels of *DC-SCRIPT *were found in breast tumors with a ductal carcinoma *in situ *(DCIS) component or infiltrating lobular carcinoma compared with infiltrating ductal carcinomas (both *P <*0.01). Of 308 LNN tumors, intrinsic subtyping data were available [20]. In these tumors, basal-like tumors had the lowest levels and normal-like breast tumors expressed significantly higher levels of *DC-SCRIPT *compared with the other intrinsic subtypes (*P *< 0.001; Figure S1 in Additional file 2). Furthermore, luminal A tumors expressed higher levels of *DC-SCRIPT *and *ESR2 *but lower levels of *ESR1 *compared with luminal B tumors (median levels in luminal A versus luminal B: 0.0078 and 0.056 for *DC-SCRIPT *[*P *= 0.003], 0.0095 and 0.0023 for *ESR2 *[*P *< 0.001], and 6.1 and 13.6 for *ESR1 *[*P *< 0.001]). This may be explained at least partly by the fact that, in this cohort of 308 LNN tumors, the luminal B tumors contained a higher percentage of invasive epithelial cells (mean ± standard deviation [SD]: 77% ± 9% for the *n *= 64 luminal B tumors versus 67% ± 12% for the *n *= 71 luminal A tumors).

### *DC-SCRIPT *and tumor aggressiveness in univariate and multivariable analyses

In the analyses including all 1,505 M0 patients, increasing levels of *DC-SCRIPT *mRNA were significantly associated with favorable DFS, MFS, and OS (HR 0.78, 0.74, and 0.77, respectively; all *P *< 0.001). To test for a relation between DC-SCRIPT mRNA levels and tumor aggressiveness (that is, the natural course of the disease without the confounding effect of systemic adjuvant therapy), we restricted our next analyses of MFS to those 837 LNN disease patients who had not received (neo)adjuvant systemic therapy. The significant relationships of *DC-SCRIPT *as a continuous variable in these univariate analyses justified the use of the previously identified cut point that dichotomized the cohort in 33.3% of the patients with low levels and 66.7% of patients with high levels of *DC-SCRIPT *mRNA in their primary tumors [7]. In univariate analysis, high levels of *DC-SCRIPT *were significantly associated with a favorable prognosis (HR 0.55; *P *< 0.001) (Table 2). When added to a multivariable base model for LNN disease - which included the traditional prognostic factors of age, menopausal status, grade, and *PGR *- stratified by *ESR1 *and tumor size to meet the proportional hazards assumption, the association of *DC-SCRIPT *with MFS remained highly significant (HR 0.60; *P <*0.001) (Table 2). Adding *ESR2 *to the model did not significantly affect the prognostic value of *DC-SCRIPT *in these analyses (Table 2).

**Table 2:** Univariate and multivariable analyses for metastasis-free survival as a function of *DC-SCRIPT *in lymph node-negative disease

		Univariate analysis				Multivariate analysis^a^			
			
Factor	Number	HR	95% CI		*P *value	HR	95% CI		*P *value
	837								
Age, years									
≤ 40	114	1				1			
41-55	295	0.88	0.63	1.22		0.95	0.67	1.35	
56-70	270	0.72	0.51	1.02		0.69	0.40	1.20	
>70	158	0.53	0.35	0.81	< 0.01	0.49	0.27	0.90	0.077
Menopausal status									
Premenopausal	350	1				1			
Postmenopausal	487	0.78	0.62	0.97	0.028	1.08	0.70	1.66	0.731
Grade									
Poor	422	1				1			
Unknown	262	1.02	0.79	1.30		1.12	0.87	1.44	
Moderate and good	153	0.49	0.34	0.71	< 0.001	0.54	0.37	0.78	< 0.001
*PGR *mRNA status^b^									
Negative, < 0.1	312	1				1			
Positive, ≥0.1	525	0.68	0.54	0.85	0.001	0.71	0.53	0.95	0.022
Tumor size									
≤ 2 cm	378	1							
>2 cm + unknown	459	1.26	1.00	1.59	0.047	Analyses stratified by tumor size to meet the proportional hazards assumption			
*ESR1 *mRNA status^b^									
Negative, < 0.2	199	1							
Positive, ≥0.2	638	0.77	0.59	0.99	0.040				
Factor analyzed						Additions to the base model			
*DC-SCRIPT*									
Continuous	837	0.77	0.67	0.88	< 0.001	0.80	0.70	0.92	0.001
33.3% low	277	1				1			
66.7% high	560	0.55	0.43	0.69	< 0.001	0.60	0.47	0.76	< 0.001
*ESR2 *mRNA status^b^									
Continuous	820	0.88	0.79	0.99	0.034	0.86	0.76	0.96	0.011
Dichotomized low, < 0.005	410	1				1.00			
Dichotomized high, ≥0.005	410	0.80	0.63	1.00	0.052	0.75	0.59	0.94	0.014
*DC-SCRIPT *and *ESR2 *combined									
Both low	183	1				1			
*DC-SCRIPT *low, *ESR2 *high	91	0.74	0.51	1.08		0.71	0.49	1.04	
*DC-SCRIPT *high, *ESR2 *low	227	0.49	0.36	0.67		0.55	0.40	0.76	
Both high	319	0.50	0.38	0.67	< 0.001	0.52	0.39	0.69	< 0.001

^a^Multivariable analyses were conducted in two blocks. First, a model including all established clinicopathological factors was fitted. The Cox proportional hazards assumptions were checked and the analyses were stratified by tumor size and *ESR1 *to meet the proportional hazards assumption. In a second block, the contributions of *DC-SCRIPT *and *ESR2 *(as continuous or dichotomized variables) were investigated. ^b^With quantitative polymerase chain reaction cut point for positive versus negative *ESR1 *and *PGR*, 0.2 and 0.1, respectively, and for *ESR2 *at the median level of 0.005 (mRNA levels relative to reference gene set). CI, confidence interval; *DC-SCRIPT*, dendritic cell-specific transcript gene; *ESR*, estrogen receptor gene; HR, hazard ratio; *PGR*, progesterone receptor gene; pT1, small tumor without lymphatic/vascular invasion.

**Figure 1:**
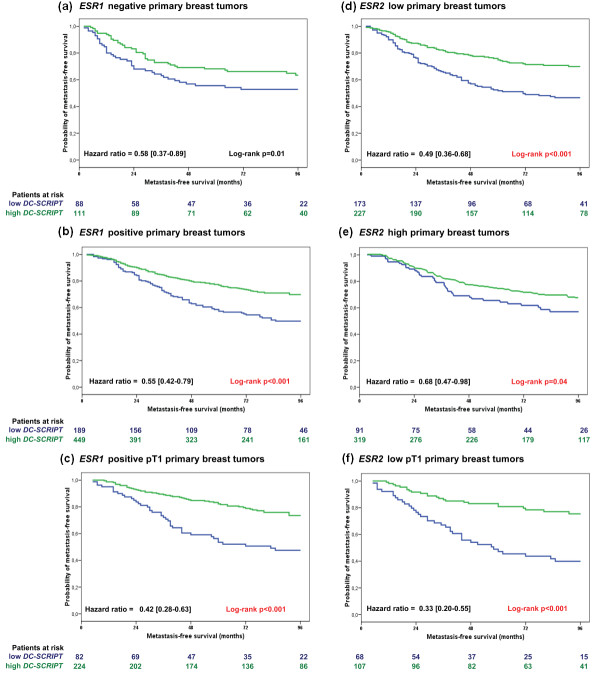
**Metastasis-free survival as a function of dichotomized *DC-SCRIPT***. Metastasis-free survival is shown as a function of dichotomized DC-SCRIPT in 837 lymph-node negative, primary breast cancer patients after subdividing them according high and low *ESR1 *and *ESR2 *in the primary tumor and tumor size. **(a) ***ESR1 *negative primary breast tumors, **(b) ***ESR1 *positive primary breast tumors, **(c) ***ESR1 *positive pT1 primary breast tumors, **(d) ***ESR2 *low primary breast tumors, **(e) ***ESR2 *high primary breast tumors, **(f) ***ESR2 *low pT1 primary breast tumors. Quantitative polymerase chain reaction cut points are shown for high versus low *DC-SCRIPT *(66.7% versus 33.3%) [7], for positive versus negative *ESR1 *(0.2) [14], and for *ESR2*-low versus -high at the median level of 0.005 (mRNA levels relative to reference gene set). Patients at risk are indicated. *DC-SCRIPT*, dendritic cell-specific transcript; *ESR*, estrogen receptor; pT1, small tumor without lymphatic/vascular invasion.

Because the proportional hazards assumptions were violated by *ESR1 *and tumor size and because DC-SCRIPT is a transcriptional co-regulator of nuclear receptors - including the, for breast cancer biologically relevant, steroid hormone receptors - we next explored its prognostic value as continuous variable in subgroups of tumors stratified by steroid hormone receptor status and tumor size (Table 3 and Figure 1). Subdividing the 837 primary LNN tumors into *ESR1*-positive and -negative [14] showed that increasing levels of *DC-SCRIPT *were, in univariate and multivariable analyses, associated with good prognosis only for the patients with *ESR1*-positive tumors. Subdividing these LNN tumors at the median level of *ESR2 *into high and low revealed that, in contrast to *ESR1*, increasing levels of *DC-SCRIPT *were, in both univariate and multivariable analyses, associated with good prognosis only for patients with primary tumors with low levels of *ESR2*. With respect to tumor size, in univariate and multivariable analyses, increasing levels of *DC-SCRIPT *were associated with good prognosis only for pT1 (small tumor without lymphatic/vascular invasion) tumors and not for larger tumors. These and additional exploratory Cox univariate analyses are summarized in Table 3. The prognostic value of *DC-SCRIPT *is visualized in Kaplan-Meier curves (Figure 1) as a dichotomized variable in these biologically relevant LNN *ESR1*-negative (Figure 1a) and -positive (Figure 1b) and LNN *ESR2*-high (Figure 1d) and -low (Figure 1e) subsets in combination with patients with pT1 primary tumors (Figure 1c, f).

**Table 3:** Disease-free survival, metastasis-free survival, and overall survival as a function of continuous *DC-SCRIPT *in lymph node-negative disease

Association with continuous *DC-SCRIPT*		Disease-free survival				Metastasis-free survival				Overall survival			
				
Cohort	Number	HR	95% CI		*P *value	HR	95% CI		*P *value	HR	95% CI		*P *value
Lymph node-negative	837	0.82	0.73	0.93	0.001	0.77	0.67	0.88	< 0.001	0.82	0.72	0.94	0.005
*ESR1 *mRNA-negative^a^	199	0.94	0.76	1.17	0.59	0.93	0.73	1.18	0.53	0.88	0.70	1.11	0.30
*ESR1 *mRNA-positive^a^	638	0.79	0.68	0.90	0.001	0.72	0.62	0.85	< 0.001	0.81	0.69	0.96	0.014
*PGR *mRNA-negative^a^	312	0.88	0.74	1.06	0.19	0.84	0.69	1.03	0.10	0.88	0.72	1.08	0.22
*PGR *mRNA-positive^a^	525	0.81	0.69	0.94	0.007	0.75	0.63	0.89	0.001	0.82	0.68	0.99	0.04
*ESR2 *mRNA-low^a^	410	0.76	0.64	0.91	0.003	0.69	0.56	0.84	< 0.001	0.73	0.64	0.97	0.026
*ESR2 *mRNA-high^a^	410	0.93	0.78	1.11	0.43	0.89	0.73	1.09	0.26	0.92	0.75	1.13	0.41
Tumor size ≤ 2 cm (pT1)^b^	378	0.74	0.61	0.89	0.001	0.67	0.54	0.83	0.000	0.73	0.59	0.91	0.005
Tumor size >2 cm^b^	459	0.92	0.79	1.08	0.31	0.86	0.72	1.03	0.10	0.91	0.76	1.09	0.31
*ESR1 *mRNA-positive, tumor size ≤ 2 cm	306	0.69	0.56	0.85	0.001	0.61	0.48	0.78	< 0.001	0.72	0.56	0.93	0.010
*ESR1 *mRNA-positive, tumor size >2 cm	332	0.91	0.75	1.10	0.34	0.84	0.68	1.05	0.13	0.90	0.72	1.14	0.39
*ESR2 *mRNA-low, tumor size ≤ 2 cm	175	0.57	0.43	0.76	< 0.001	0.51	0.37	0.70	< 0.001	0.60	0.44	0.83	0.002
*ESR2 *mRNA-high, tumor size >2 cm	218	0.98	0.78	1.23	0.84	0.91	0.71	1.18	0.49	0.93	0.74	1.21	0.58
*ESR1*-positive and *ESR2*-low, tumor size ≤ 2 cm	147	0.63	0.45	0.87	0.005	0.54	0.38	0.78	< 0.001	0.63	0.43	0.92	0.017
*ESR1*-positive and *ESR2*-low, tumor size >2 cm	181	0.94	0.71	1.24	0.66	0.94	0.68	1.29	0.69	1.03	0.73	1.45	0.89
*ESR1*-positive or *ESR2*-low or both, tumor size ≤ 2 cm	334	0.65	0.53	0.79	< 0.001	0.57	0.46	0.71	< 0.001	0.67	0.53	0.84	0.001
*ESR1*-positive or *ESR2*-low or both, tumor size >2 cm	386	0.90	0.76	1.08	0.25	0.81	0.66	0.99	0.037	0.87	0.71	1.07	0.20

^a^With quantitative polymerase chain reaction cut point for positive versus negative *ESR1 *and *PGR*, 0.2 and 0.1, respectively, and for *ESR2 *at the median level of 0.005 (mRNA levels relative to reference gene set). ^b^Interaction with continuous *DC-SCRIPT *(*P *< 0.05). CI, confidence interval; *DC-SCRIPT*, dendritic cell-specific transcript gene; *ESR*, estrogen receptor gene; HR, hazard ratio; *PGR*, progesterone receptor gene; pT1, small tumor without lymphatic/vascular invasion.

### *DC-SCRIPT *and response to first-line endocrine therapy

*DC-SCRIPT *expression levels were evaluated in 296 hormone-naïve ER-positive primary breast tumors from patients whose relapse was treated with first-line tamoxifen monotherapy. These patients had not received (neo)adjuvant endocrine systemic treatment. In univariate analyses, no statistically significant associations were observed between *DC-SCRIPT *as transformed continuous variable and PFS or clinical benefit after start of first-line treatment with tamoxifen (HR = 1.08 [0.99 to 1.18], *P *= 0.07 and odds ratio = 0.88 [0.74 to 1.05], *P *= 0.16, respectively).

## Discussion

DC-SCRIPT has been identified as a key modulator of nuclear receptor activity that has prognostic value in breast cancer [7]. The clinical conclusions about *DC-SCRIPT *mRNA expression as a prognostic marker in breast cancer were based on non-randomized retrospective analyses in three small, breast cancer cohorts from Nijmegen (The Netherlands) and still required independent validation. In this study, we provide a higher level of evidence as we confirm that mRNA expression values of *DC-SCRIPT *indicate outcome in an independent retrospective cohort of 1,505 primary breast cancers from Rotterdam. In addition, we confirm that *DC-SCRIPT *mRNA expression is a pure prognostic marker as it indicates - independently of current clinical prognostic markers such as age, menopausal status, grade, tumor size, and receptor status - the occurrence of distant metastasis in patients who did not receive any adjuvant systemic treatment. Because we used mRNA extracted from tumor tissue and a different mRNA isolation method (RNA-B versus column-based), an independent real-time PCR assay to detect *DC-SCRIPT*, a different type of machine to amplify the transcript, and personnel from another institute, we consider *DC-SCRIPT *a robust prognostic marker for patients with early breast cancer. The patients described in this retrospective study entered the clinic during 1978 to 2000. During this period, adjuvant therapy was not as widespread as it is nowadays; this circumstance was at the same time the strength of our cohort for the evaluation of a prognostic marker. The data that emerged from this study thus validate the hypothesis that *DC-SCRIPT *is associated with good prognosis in early disease and support the idea that DC-SCRIPT acts as a tumor suppressor in breast cancer progression [7].

Because of the size of this cohort and the biological function of DC-SCRIPT as a nuclear receptor co-regulator, we were able to include additional subgroup analyses to extend our insights into the clinical behavior and relevance of measuring *DC-SCRIPT *in primary breast cancers. High levels of *DC-SCRIPT *mRNA in primary tumors of breast cancer patients were significantly related with tumor characteristics that are associated with good prognosis, such as DCIS, infiltrating lobular carcinoma, breast tumors of the normal-like and luminal A subtype, and small (pT1), well-differentiated, steroid hormone receptor-positive tumors. While *ESR1 *is localized mainly in tumors with at least 70% invasive epithelial cells (*P *< 0.001), we showed for both *ESR2 *and *DC-SCRIPT *a positive correlation with tumors with less than 70% invasive epithelial cells (*P *< 0.001). As normal epithelial cells in tumors with less than 70% invasive epithelial cells express the highest levels of *DC-SCRIPT*, they could be responsible for this correlation. Furthermore, infiltrating leukocytes in the stroma might have contributed to the detected signal [4,5]. Alternatively, or additionally, stromal cells may have played a role in the induction of *DC-SCRIPT *in the epithelial tumor cells. In analogy, *ESR2 *is - apart from breast cancer epithelial tumor cells - also expressed in adjacent infiltrating lymphocytes, fibroblasts, and endothelial cells [3].

Interestingly, in tumors that express relatively high *ESR2 *mRNA levels and that in general have a higher stromal content, *DC-SCRIPT *expression has little or no prognostic value. Thus, while in early *ESR1*-positive breast cancer *DC-SCRIPT *inhibits progression of breast cancer, this effect appears to be neutralized in tumors high in *ESR2*. Indeed, *ESR2 *has been reported to be dominant over *ESR1 *and able to counteract the proliferation-inducing activities of *ESR1 *[1,2]. Unraveling the precise role of *DC-SCRIPT *in the complex genomic and non-genomic interplay between *ESR1*, *ESR2*, and their isoforms [21-23] might turn out to be rewarding for elucidating the 'yin-yang' role of these factors in breast cancer.

As DC-SCRIPT can inhibit ERα and PR activity, a second aim of the study was to address whether DC-SCRIPT affects the response to endocrine therapy. In our previous study, we had already explored the value of *DC-SCRIPT *mRNA expression to indicate outcome in a cohort of breast cancer patients who received adjuvant tamoxifen [7]. However, in the adjuvant setting - that, for ethical reasons, nowadays includes only non-randomly assigned patients among treated and untreated arms - one cannot discriminate between tumor aggressiveness and response to treatment [24]. The current retrospective study included hormone-naïve patients (that is, not having received any [neo]adjuvant endocrine treatment) who received first-line tamoxifen treatment for their advanced disease and therefore was better suited to study a putative relation of *DC-SCRIPT *and response to therapy. Despite the positive association of *DC-SCRIPT *with *ESR1*, *DC-SCRIPT *levels were unable to identify patients with *ESR1*-positive primary tumors at high or low risk to progress if treated with tamoxifen. Thus, although DC-SCRIPT can modulate the activity of *ESR1*, it does not affect the response to endocrine therapy with tamoxifen in advanced breast cancer. The early loss of *DC-SCRIPT *during cancer progression might explain this absence of a response in the metastatic disease setting.

## Conclusions

This independent retrospective quantitative RT-PCR study validates that high levels of *DC-SCRIPT *are associated with reduced tumor aggressiveness. The association is particularly strong for small tumors with high *ESR1 *or low *ESR2 *mRNA levels or both. Finally, although DC-SCRIPT negatively regulates *ESR1 *and *PGR *activity, *DC-SCRIPT *levels measured in the primary tumors are not associated with response to first-line endocrine treatment for advanced disease. This finding is in line with DC-SCRIPT as an early marker for disease.

## Abbreviations

*B2M*: beta-2-microglobulin gene; bp: base product; Ct: cycle threshold; DCIS: ductal carcinoma *in situ*; DC-SCRIPT: dendritic cell-specific transcript; DFS: disease-free survival; ER: estrogen receptor; *ESR*: estrogen receptor gene; *HMBS*: hydroxymethylbilane synthase gene; *HPRT1*: hypoxanthine guanine phosphoribosyltransferase 1 gene; HR: hazard ratio; LNN: lymph node-negative; M0: no metastasis; M1: with metastasis; MFS: metastasis-free survival; OS: overall survival; PCR: polymerase chain reaction; PFS: progression-free survival; *PGR*: progesterone receptor gene; PPAR: peroxisome proliferator-activated receptor; pT1: small tumor without lymphatic/vascular invasion; PR: progesterone receptor; RAR: retinoic acid receptor; RT-PCR: reverse transcriptase polymerase chain reaction; SYBR: N',N'-dimethyl-N-[4-[(E)-(3-methyl-1,3-benzothiazol-2-ylidene)methyl]-1-phenylquinolin-1-ium-2-yl]-N-propylpropane-1,3-diamine.

## Competing interests

The authors declare that they have no competing interests.

## Authors' contributions

AMS participated in the study design, collected laboratory data on the patients, performed laboratory work and statistical analyses, and wrote the manuscript. MA participated in the study design, performed laboratory work, and provided critical revision of the manuscript. MPL collected laboratory data on the patients, performed the clinical statistical analyses, and provided critical revision of the manuscript. PNS provided critical revision of the manuscript and participated in the study design. VdW and AvG performed the laboratory work and provided critical revision of the manuscript. JAF and JWMM participated in the study design, provided the study material and clinical information, and provided critical revision of the manuscript. GJA participated in the study design and provided critical revision of the manuscript. All authors read and approved the final manuscript.

## Supplementary Material

Additional file 1 **Supplementary materials and methods**. A word file containing additional Materials and methods [25-28]. Click here for file

Additional file 2 **Figure S1 - *DC-SCRIPT *mRNA expression in breast cancer subtypes**. The box-plot shows the five statistics (lower whisker is 5% minimum, lower box part is 25^th ^percentile, solid line in box presents the median, upper box part is 75^th ^percentile and upper whisker is 95% maximum). Figure depicts *P *for Mann-Whitney U test to identify significantly different expression of *DC-SCRIPT *in between subtypes. Click here for file
