# Supplementary material for: Clinical significance of the nuclear receptor co-regulator DC-SCRIPT in breast cancer: an independent retrospective validation study
Source: Breast Cancer Res. 2010 Dec 1;12(6):R103. doi: 10.1186/bcr2786 (PMC3046448; doi:10.1186/bcr2786)
Supplement: Additional file 1 — Supplementary materials and methods. A word file containing additional Materials and methods [25-28]. [file bcr2786-S1.DOC]

***Online Only* Information, additional Materials and methods [additional file 1]**

**Patient samples**

Frozen tumor samples were originally submitted to our reference laboratory from regional hospitals for measurements of steroid hormone receptors and have been stored in our liquid nitrogen tumor bank at the Erasmus Medical Center (Rotterdam, The Netherlands). Guidelines for primary treatment were similar for all hospitals. All available frozen tumor specimens from female patients with breast cancer who entered the clinic during 1978–2000 and from whom detailed clinical follow-up was available[19, 25], were processed for mRNA analysis. Further inclusion criteria were: >100 mg frozen tissue available, invasive breast cancer, no previous other cancer (except basal cell skin cancer or early-stage cervical cancer stage Ia/Ib), no second contralateral primary breast tumor as first relapse for first-line studies, at least 30% invasive tumor cell nuclei, and good RNA quality with both the 18S and 28S bands visible on gel. Thirty percent of the samples were rejected because of a too low percentage tumor cells and/or poor RNA quality and/or because the RNA yield was less than 2 ug.

The primary breast tumors used for this study were from patient with detailed clinical follow-up information as described previously[12-14, 26]. Fifteen hundred and five patients were included in the prognosis analyses. Of these 1,505 M0 patients 264 were included for the analysis of recurrent disease combined with an additional 32 M1 patients. All 1,537 patients were treated with breast conserving lumpectomy (44%) or modified mastectomy (56%). Axillary dissection was performed in most cases (99%). Adjuvant radiotherapy was given to 98% and 46% of the patients undergoing lumpectomy or modified mastectomy, respectively, and 379 (25%) of all patients received adjuvant radiation at the nodes. ER status was determined by routine ligand-binding assays or enzyme immunoassays. Pathological review was performed in the various participating regional hospitals as described previously[15].

For the analysis of MFS, 1,505 patients, of whom 837 LNN, were included. None of the 837 LNN patients received systemic adjuvant therapy.

Patients were evaluated every three months for the first 2 years, every 6 months for the next 3 years and once a year thereafter. Median follow-up time of patients alive was 90.3 months. Of the 837 LNN patients 383 had a disease relapse, 300 developed a distant metastasis and 273 had died during follow-up.

For the evaluation of PFS and clinical benefit after start of first-line tamoxifen for metastatic disease, 296 patients with ER protein-positive primary breast tumors (264/1,505 of whom 127/837 patients LNN were also included in the analysis of MFS and experienced a relapse) who received tamoxifen monotherapy as first-line treatment for recurrent disease were included[16]. Basic characteristics of these patients at diagnosis were: median age at diagnosis 58 years (range 26 to 89) and 61 (29 to 90) at start of therapy of whom 35% were pre-menopausal at diagnosis and 26% at start of therapy; 44% node negative; grade poor (54%), good/moderate (13%), or unknown (33%). None of these patients had received prior adjuvant hormonal therapy and 19% had received prior adjuvant chemotherapy (21 patients anthracycline-based ((F)AC/FEC) and 34 patients non-anthracycline-based (CMF)).

Patients were routinely followed at the outpatient clinic, generally once every 3 weeks during the first 6 months, and in case of objective response approximately every 6 weeks[27, 28]. All participating hospitals used the same guidelines for disease detection and progression, however, subtle differences may have existed due to the interpretation by individual physicians. Skin metastases were assessed clinically by palpation, measured and documented by photography; lymph node metastases were assessed by palpation or when necessary by ultrasound; lung metastases were routinely followed by X-thorax (once every 6-12 weeks), and by CT-thorax where applicable; liver metastases were always followed by ultrasound or CT of the liver, in general once every 12 weeks; brain metastases were assessed by CT or magnetic resonance imaging if indicated; bone metastases were followed by X-rays (every 6-12 weeks) and bone scan (every 6-12 months) as a standard, and by magnetic resonance imaging if indicated. Furthermore, plasma tumor marker levels (CA15.3 and/or CA125) were measured regularly.

The type of response to therapy was recorded as defined by standard International Union Against Cancer criteria[18]. For the patients treated with first line tamoxifen, no clinical benefit occurred in 111 patients (94 progressive disease (i.e., 25% or more increase) and 17 stable disease shorter or equal than 6 months). Clinical benefit of first-line tamoxifen treatment was observed in 185 patients, of whom 12 showed a complete remission (i.e., complete disappearance of all metastases), 39 a partial remission (i.e., at least 50% reduction) and the remaining 134 patients experienced stable disease for longer than 6 months. Median follow-up time for treatment of recurrent disease was 38 (4 to 120) months. Two-hundred nineteen patients had died at the end of the follow-up.

**Tissue Processing**

Primary tumor tissue was processed as described previously[16]. In summary, 20 to 60 cryostat sections of 30 µm, corresponding to 30 to 100 mg, were cut from snap-frozen tissues for RNA isolation. To assess the amount of invasive tumor cell nuclei relative to the amount of surrounding stromal cells, 5 µm sections were cut for hematoxylin and eosin staining, before, in between, and after cutting the sections for RNA isolation. For this study, only specimen with at least 30% invasive tumor cell nuclei, distributed uniformly over at least 70% of the section area, were included.
