# Supplementary figures and images for: Clinical significance of the nuclear receptor co-regulator DC-SCRIPT in breast cancer: an independent retrospective validation study
Source: Breast Cancer Res. 2010 Dec 1;12(6):R103. doi: 10.1186/bcr2786 (PMC3046448; doi:10.1186/bcr2786)

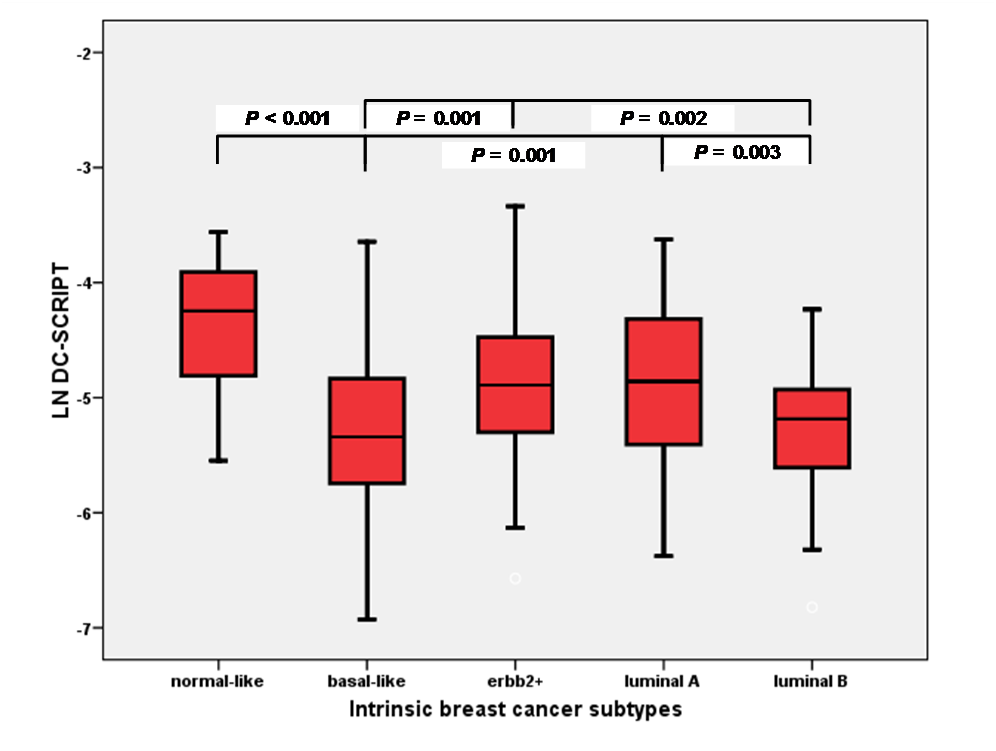

Supplement: Additional file 2 — Figure S1 - DC-SCRIPT mRNA expression in breast cancer subtypes. The box-plot shows the five statistics (lower whisker is 5% minimum, lower box part is 25th percentile, solid line in box presents the median, upper box part is 75th percentile and upper whisker is 95% maximum). Figure depicts P for Mann-Whitney U test to identify significantly different expression of DC-SCRIPT in between subtypes. [file bcr2786-S2.TIFF]
